# Genomic surveillance of SARS-CoV-2 using long-range PCR primers

**DOI:** 10.3389/fmicb.2024.1272972

**Published:** 2024-02-14

**Authors:** Sangam Kandel, Susanna L. Hartzell, Ashton K. Ingold, Grace A. Turner, Joshua L. Kennedy, David W. Ussery

**Affiliations:** ^1^Department of Biomedical Informatics, University of Arkansas for Medical Sciences, Little Rock, AR, United States; ^2^Arkansas Children's Research Institute, Little Rock, AR, United States; ^3^Department of Pediatrics, University of Arkansas for Medical Sciences, Little Rock, AR, United States; ^4^Department of Internal Medicine, University of Arkansas for Medical Sciences, Little Rock, AR, United States

**Keywords:** SARS-CoV-2, surveillance, nanopore, long-range primers, sequencing, genomic epidemiology

## Abstract

**Introduction:**

Whole Genome Sequencing (WGS) of the SARS-CoV-2 virus is crucial in the surveillance of the COVID-19 pandemic. Several primer schemes have been developed to sequence nearly all of the ~30,000 nucleotide SARS-CoV-2 genome, using a multiplex PCR approach to amplify cDNA copies of the viral genomic RNA. Midnight primers and ARTIC V4.1 primers are the most popular primer schemes that can amplify segments of SARS-CoV-2 (400 bp and 1200 bp, respectively) tiled across the viral RNA genome. Mutations within primer binding sites and primer-primer interactions can result in amplicon dropouts and coverage bias, yielding low-quality genomes with ‘Ns’ inserted in the missing amplicon regions, causing inaccurate lineage assignments, and making it challenging to monitor lineage-specific mutations in Variants of Concern (VoCs).

**Methods:**

In this study we used a set of seven long-range PCR primer pairs to sequence clinical isolates of SARS-CoV-2 on Oxford Nanopore sequencer. These long-range primers generate seven amplicons approximately 4500 bp that covered whole genome of SARS-CoV-2. One of these regions includes the full-length S-gene by using a set of flanking primers. We also evaluated the performance of these long-range primers with Midnight primers by sequencing 94 clinical isolates in a Nanopore flow cell.

**Results and discussion:**

Using a small set of long-range primers to sequence SARS-CoV-2 genomes reduces the possibility of amplicon dropout and coverage bias. The key finding of this study is that long range primers can be used in single-molecule sequencing of RNA viruses in surveillance of emerging variants. We also show that by designing primers flanking the S-gene, we can obtain reliable identification of SARS-CoV-2 variants.

## 1 Introduction

Whole Genome Sequencing (WGS) is widely used for the surveillance of Severe Acute Respiratory Syndrome Coronavirus-2 (SARS-CoV-2), the causative agent of the pandemic disease COVID-19 (Huang et al., 2020; Wu et al., 2020; Zhou et al., 2020). At the time of writing (August, 2023), there are more than 15.8 million genomes available in the GISAID database^1^ and more than 8.1 million genomes in GenBank.^2^ Sequencing SARS-CoV-2 genomes is crucial in tracking viral mutations that can affect viral transmission (Kupferschmidt and Wadman, 2021; Brito et al., 2022; Escalera et al., 2022; Carabelli et al., 2023), disease pathogenesis (Bakhshandeh et al., 2021), vaccine efficacy (Hoffmann et al., 2021; Madhi et al., 2021; Chatterjee et al., 2023), and virulence (Issa et al., 2020; Carabelli et al., 2023). A variety of methods, including metagenomic sequencing, hybridization capture, direct RNA sequencing, and target enrichment using multiplex PCR have been used for sequencing SARS-CoV-2 (Carbo et al., 2020; Charre et al., 2020; Deng et al., 2020; Wu et al., 2020; Xiao et al., 2020; Butler et al., 2021; Liu et al., 2021; Rehn et al., 2021; Gerber et al., 2022; Vacca et al., 2022). Most of the target enrichment methods require reverse transcription to generate a double-stranded cDNA copy of the genomic RNA (gRNA) and then utilize this cDNA as a template for DNA sequencing, using multiplex primers to cover the whole genome of SARS-CoV-2 (Grubaugh et al., 2019).

**Figure 1:**
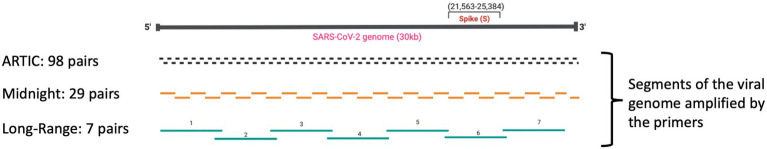
Comparison of ARTIC, Midnight, and Long-Range PCR primers.

Target enrichment using PCR amplicons and subsequent Oxford Nanopore Sequencing is extremely popular and relatively inexpensive (~$10 per sample), with a quick turnaround time (~24 h from sample to GenBank file). Target enrichment using publicly available ARTIC Network PCR primers (Quick 2020; Tyson et al., 2020), Entebbe primers (1.5 kb-2Kb) (Cotten et al., 2021), MRL primers (1.5 kb-2.5 kb) (Arana et al., 2022), and Midnight Primers (Freed et al., 2020) are used to sequence SARS-CoV-2 with Oxford Nanopore flow cells. Among these primer schemes, ARTIC primers and Midnight primers are the most used to sequence clinical isolates of SARS-CoV-2 (Table 1; Figure 1). ARTIC primers V4 includes 98 primer pairs, each amplifying ~400 bp fragments along the viral genome, which can be sequenced on either Illumina or Oxford Nanopore platforms. The ‘Midnight primers’ have 29 primer pairs that generate amplicons with a targeted size of 1,200 base pairs, taking advantage of the longer read lengths of third-generation sequencing, including Oxford Nanopore flow cells. Generation of full-length high-quality consensus sequences depends upon the quality and quantity of the viral load in clinical samples, as well as the mutations occurring within the primer binding regions of the viral genome (Davis et al., 2021; Liu et al., 2021; Kuchinski et al., 2022). Amplicon dropouts and coverage bias at different amplicon regions have been observed with the sequencing protocols based on ARTIC (Itokawa et al., 2020; Kuchinski et al., 2022) as well as Midnight primers (Bei et al., 2022; Kuchinski et al., 2022). Mutations within the primer binding site can prevent primer-annealing and result in ‘dropout’ or loss of that amplicon, leading to incomplete genome sequences (Sanderson and Barrett, 2021; Bei et al., 2022). Furthermore, primer-primer interactions could result in amplification bias of interacting amplicons (Itokawa et al., 2020), resulting in coverage bias and affecting the identification of mutations in the viral genome that are key in the nomenclature of emerging variants.

**Table 1:** Comparison of ARTIC, Midnight, and Long-range primers used to sequence SARS-CoV-2 clinical isolates.

	ARTIC	Midnight	Lon-range
Number of primers sets	98	29	7
Amplicon size (base pairs)	400	1,200	4,500

The variants of SARS-CoV-2 are determined by a combination of several mutations that occur mainly within the Spike gene. For example, in the Alpha variant (B.1.1.7), there are 14 critical lineage-defining mutations within the S gene (Galloway et al., 2021). Similarly, Omicron subvariant B.1.1.529 has 60 mutations within the viral genome, including 15 key mutations within the receptor binding domain (He et al., 2021). The characteristic mutation within the S gene for the Alpha variant B.1.1.7 (Meng et al., 2021; Clark et al., 2022) and the Omicron variants B.1.1.529, BA.1, BA.1.1 (Clark et al., 2022) is the deletion of two amino acids at positions 69 and 70 (del H69/V70)^3^. This deletion inhibits the PCR amplification of the S-gene (S-Gene Target Failure, or SGTF) in diagnostic PCR assays such as the ThermoFisher TaqPath™ COVID-19 Combo Kit RT-PCR (Davies et al., 2021; Clark et al., 2022) that targets the N, ORF1ab, and S gene regions. This deletion (del H69/V70) results in a false-negative result for the S-gene targeted diagnostic test. SGTF became a proxy for early detection of Alpha and Omicron B.1.1.529 variants (Galloway et al., 2021). In addition, a mutation at position 27,807 (Cytosine substituted to Thymine) within amplicon 28, also a primer annealing site (Primer 28_LEFT, pool B of Midnight primer) (Supplementary Figure 1), caused a common dropout in the Delta variant genome when using Midnight Primers (Kuchinski et al., 2022). Spiking Primer pool B with a custom primer designed by substituting Cytosine with Thymine base not only corrected the dropout but also increased the coverage at this region (Constantinides et al., 2022). Furthermore, the genome sequences of two BA.2 Omicron variants from Arkansas (GenBank Accession: OM863926, ON831693) sequenced using Midnight Primers in Oxford Nanopore GridION have a complete dropout at amplicon region 21 (20,677-21,562). The Omicron and the Alpha variant waves taught us that tests and primers designed toward regions within the S gene could result in false-negative tests because this gene encodes a surface protein, subjecting it to varying selectional pressures (Julenius and Pedersen, 2006). Variations can lead to problems that are troublesome in deciding the public health interventions needed to control the transmission and spread of COVID-19 disease.

Multiplex primers used to sequence SARS-CoV-2 viral isolates must be targeted to bind regions that are conserved with little variance to avoid dropout failures secondary to the primers not binding. Long-range PCR primers targeting the amplification of 4,500 bp can prevent the ‘S-gene dropouts’, as the primer binding sites flanking the S-gene region are located within highly conserved regions on either side of the S gene. The S gene is approximately 3,822 base pairs long and stretches between the nucleotide position 21,563 to 25,384 along the viral genome. Therefore, these long-range PCR primers can generate amplicons around 4,500 bp that will cover the entire S gene, making the chances of amplicon dropout within the S-gene minimal. We have previously demonstrated whole-genome cDNA sequences from Mumps genomes using long-range PCR yielding fragments of ~5,000 bp in length from buccal samples (Alkam et al., 2019). In addition to our work, long-range semi-nested PCR have been used to sequence Ebola virus (Seifert et al., 2018), Middle East respiratory syndrome coronavirus (MERS-CoV) (Seifert et al., 2021), Hendra virus (HeV), Nipah virus (NiV) and Cedar virus (CedPV) (Yinda et al., 2020) on an Oxford Nanopore MinION sequencer. More recently, long-range primers were used to sequence clinical isolates of Monkeypox virus (MPXV) generating amplicons around 5,000 base pairs (Isabel et al., 2023) to sequence the much larger DNA viral genome of approximately 200,000 bp. We have previously identified conserved regions withing the SARS-CoV-2 genome, including regions that flank the S gene (Wassenaar et al., 2022). In this study, we designed long-range PCR primers to target these conserved areas flanking the S gene and to sequence SARS-CoV-2 isolates. Our objective was to improve the quality of the sequences generated and minimize the amplicon dropouts, as the designed primers are outside the highly variable regions.

## 2 Methods

### 2.1 Primer design

A total of 7,046 Omicron sub-variant genomes (BA.2, BA.3, BA.4, BF.5, BA.5.1, BA.5.2.1, BA.5.2) were downloaded from GISAID on 12 August, 2022. Pangolin v4.0.6 (O'Toole et al., 2021) was used to assign lineages to the genomes, and any ‘unclassified’ genomes were removed. Genome sequences that were 100% identical were then filtered out to avoid redundancy, and genome sequences having gaps of 5Ns or more in their sequences were removed that resulted in a set of 1,205 high-quality genomes that were used for multiple sequence alignment using MAFT (Katoh et al., 2019). MSA Viewer^4^ was used to visualize the alignment, and consensus sequences were downloaded from MSA Viewer. PrimalScheme (Quick et al., 2017) was used to generate primer schemes using the consensus genome generated from the alignment of 1,205 high-quality genomes, including different sub-variants of Omicron. Primers were designed using the PrimalScheme tool using the command line (Table 2): primalscheme multiplex <fasta-file> -a 4500 –o <path-to-output> -n <primers_name> -t 30 -p -g


**Table 2:** List of 7 primer pairs designed using PrimalScheme.

Name	Pool	Sequence (5ʹ-3ʹ)	Size	GC%	Tm (use 65)
1_LEFT	1	GCTTAGTGCACTCACGCAGT	20	55	61
1_RIGHT	1	ACCGAGCAGCTTCTTCCAAA	20	50	60
2_LEFT	2	AACCACTTACCCGGGTCAGG	20	60	62
2_RIGHT	2	ACTGCAGCAATCAATGGGCA	20	50	61
3_LEFT	1	CATGACACCCCGTGACCTTG	20	60	61
3_RIGHT	1	TGTAGACGTACTGTGGCAGC	20	55	60
4_LEFT	2	AGGGCCAATTCTGCTGTCAA	20	50	60
4_RIGHT	2	ATCAACAGCGGCATGAGAGC	20	55	61
5_LEFT	1	ACGTGAAGTGCTGTCTGACAG	21	52	61
5_RIGHT	1	TTCGCGTGGTTTGCCAAGAT	20	50	61
6_LEFT	2	CTACGGGTACGCTGCTTGTC	20	60	61
6_RIGHT	2	GTATCGTTGCAGTAGCGCGA	20	55	61
7_LEFT	1	GAAATTGACCGCCTCAATGAGG	22	50	61
7_RIGHT	1	CCCATCTGCCTTGTGTGGTC	20	60	61

Primers were ordered from Integrated DNA Technology (IDT) (Coralville, IA) in lab-ready form. Individual primers in each pool were mixed and resuspended to a final concentration of 100 μM. Each primer was normalized to 3 nmol during synthesis. Primers were diluted in Nuclease-free water (Sigma) to use in a final concentration of 10 μM.

High-quality genomes were downloaded from GenBank, and a consensus sequence was generated using the most recent dominant variants of SARS-CoV-2 from GenBank collected between December 2022 and March 2023. Quality filtering was done to include only those genomes that did not contain any non-ATCGN bases and those that did not have any ‘N’s in the genome sequence. The consensus sequence from this set of genomes was used to manually design the alternative primers, including amplicons 5, 6, and 7.

### 2.2 Primer analysis

MFEprimer (Wang et al., 2019) was used to predict the various quality metrics of the primer scheme designed using PrimalScheme. This method predicted that forward primer for amplicon region 5 and forward primer for amplicon region 7 could form self-dimers. Self-dimers could prevent primer annealing to the template and hence prevent the amplification of the targets resulting in the drop of amplicon. However, the experimental results suggested a high coverage at all amplicon regions. Based on our experimental results, we can conclude that primers 5 and 7 worked well.

### 2.3 Detection and quantification of SARS-CoV-2 viral mRNA

All the samples used in this study were collected at Arkansas Children’s Hospital and the University of Arkansas for Medical Sciences as routine surveillance between (November 2022 and Jan 2023). Nasal swab samples were collected in a 3 mL M4RT transport media (Remel, San Diego, CA). Samples were tested for the SARS-CoV-2 using the Aptima® SARS-CoV-2 (Panther® System, Hologic, San Diego, CA) nucleic acid amplification assay. Positive samples were stored frozen at −80°C until they could be further processed.

### 2.4 RNA extraction, library preparation, and whole genome sequencing

**Figure 2:**
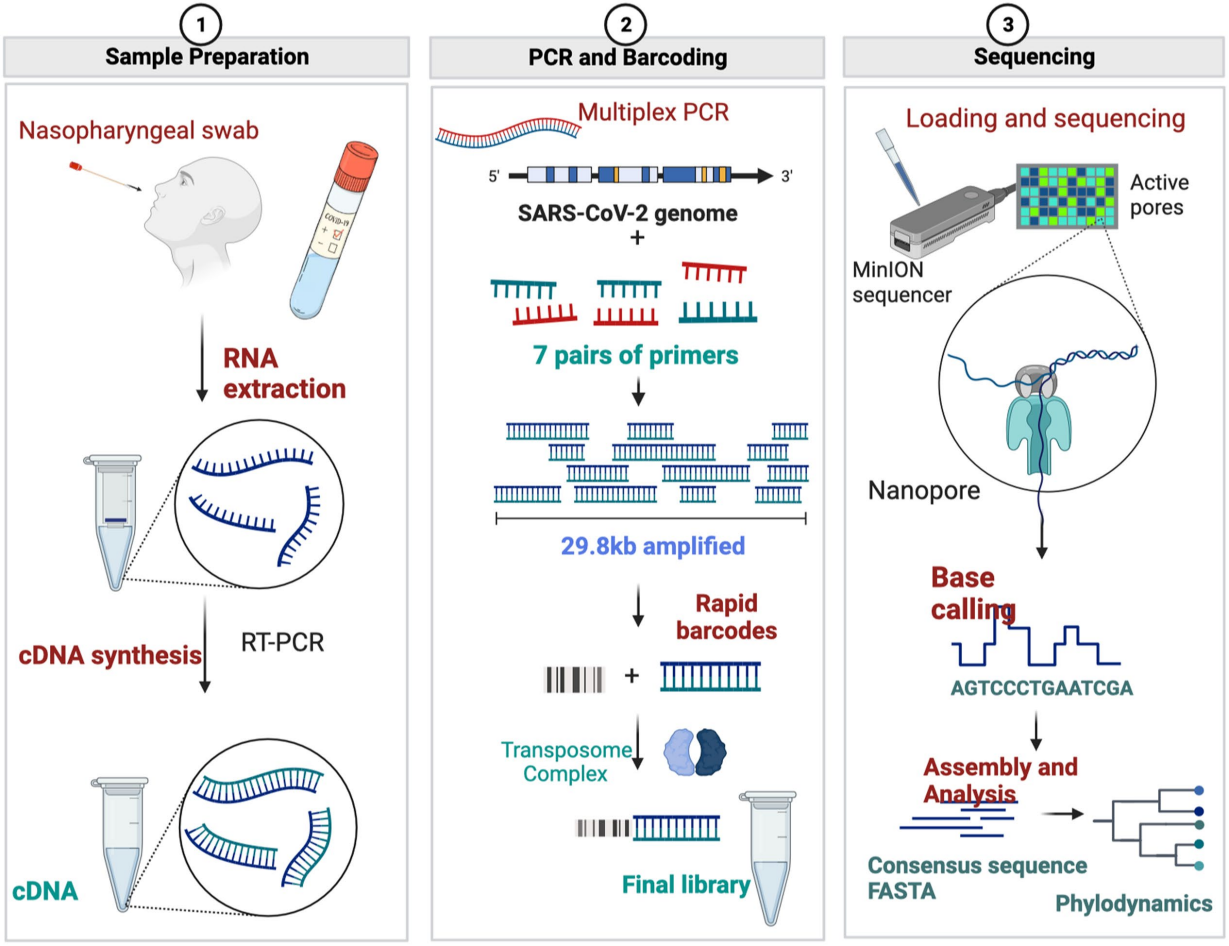
Diagrammatic representation of Oxford Nanopore Sequencing of SARS-CoV-2 using long-range PCR primers. (Figures made using BioRender.com).

A summary of the sequencing protocol is described in Figure 2. Two hundred fifty microliters of viral transport media from clinical nasal swabs were used for viral RNA extraction using the MagMax Viral/Pathogen Nucleic Isolation Kit (Applied Biosystems) on the Kingfisher Flex automated instrument (Thermofisher). Viral RNA was reverse transcribed to generate cDNA using LunaScript RT SuperMix (NEB #E3010) as described (Freed et al., 2020). Each reverse transcription reaction contained 8 μL template RNA and 2 μL LunaScript RT SuperMix (NEB #E3010). The reaction condition for reverse transcription was: 25°C for 10 min, followed by 50°C for 10 min and 85°C for 5 min. Subsequent cDNA amplification and sequencing were done using a modified sequencing protocol (Quick 2020; Table 3). In brief, viral cDNA was used in the tiling PCR method to amplify the SARS-CoV-2 viral genome using long-range PCR primers in 2 reaction pools. These primers generate PCR amplicons of around 4,500 bp size. Pool A consisted of the primers specific to amplicon regions 1, 3, 5, and 7, whereas Pool B consisted of the primers specific to amplicon regions 2, 4, and 6. A 25 μL PCR reaction mixture contained 2.5 μL template cDNA, 8.9 μL RNase-free water, 1.1 μL Primer pool A or Primer pool B (10 μM), 12.5 μL Q5 Hot Start HF 2x Master Mix (NEB # M0494X). The PCR conditions used were: 98°C for 30 s (Initial denaturation), 40 cycles of: 98°C for 10 s (Denaturation), 65°C for 30 s followed by 72°C for 5 min (Annealing and extension), and a final extension of 72°C for 5 min. Pool 1 and Pool 2 amplicons were pooled together, and 7.5 μL of each sample were barcoded using 2.5 μL of rapid barcodes available with the kit SQK-RBK004 (ONT). Barcoded samples were pooled together and cleaned using 0.8 X AMPure beads (Beckman Coulter, USA) to retain larger DNA fragments. The sequencing library was prepared using sequencing kit SQK-RBK004 (ONT), loaded onto a MinION flow cell (ONT), and sequenced for 28 h using a Minion R9.4.1 flow cell on GridION with the MinKNOW application.

**Table 3:** Optimized PCR conditions for cDNA amplification to sequence SARS-CoV-2 clinical isolates.

	Temperature	Time	Cycles
Initial denaturation	98°C	30 s	1
Denaturation	98°C	10 s	40
Annealing and extension	65°C	30 s	
	72°C	5 min	
Final extension	72°C	5 min	1
Hold	4°C	∞	

### 2.5 Bioinformatics analysis

Basecalling and demultiplexing the sequencing reads in FAST5 format was done in real-time using Guppy v5.0.7 (Wick et al., 2019) with a high-accuracy model. A minimum quality score of 9 was used to remove low-quality bases. Demultiplexed FASTQ files were processed using the ARTIC Network Bioinformatics pipeline^5^. Sequencing reads were quality filtered using artic gupplyplex method, and reference-based genome assembly was done using medaka from the artic minion method of the ARTIC bioinformatics pipeline. ONTdeCIPHER (Cherif et al., 2022) was used for generating visualization plots for genome coverage at different amplicon regions. The consensus sequence was generated by mapping to NC_045512.2 as a reference. Read depth was calculated using samtools depth (Li et al., 2009). Pangolin v4.0.6 was used to assign lineages to the genomes sequenced (O'Toole et al., 2021). Nextclade (Aksamentov et al., 2021) was used for assigning lineage as well as visualization and comparison of mutations within the viral genome.

## 3 Results

**Figure 3:**
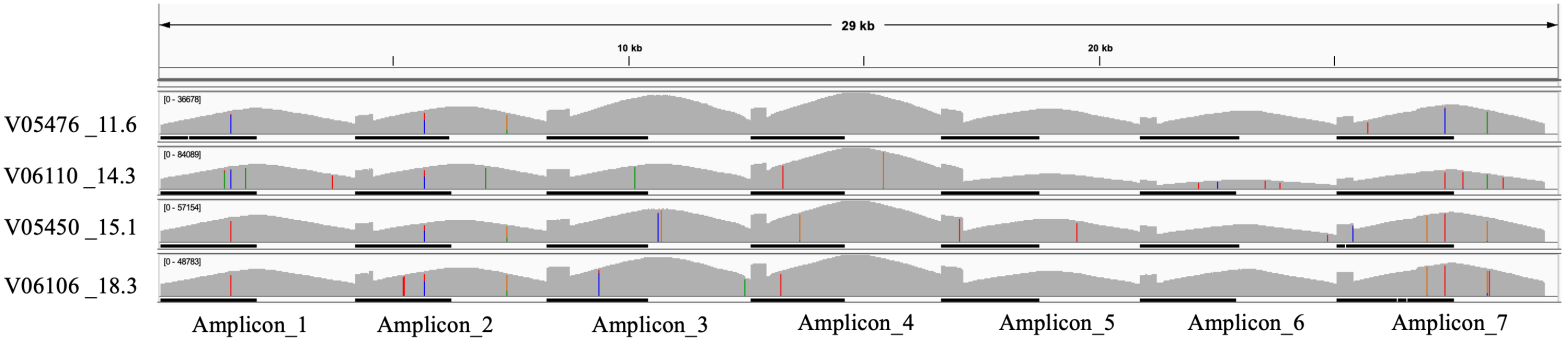
IGV plot showing seven different amplicons mapped to the SARS-CoV-2 reference genome for four samples with low CT values.

Long-range primers were used to sequence a set of four samples, with various cycle threshold (CT) values, on an Oxford Nanopore GridION machine. A lower cycle threshold is associated with higher levels of the virus in the sample, requiring fewer amplification cycles for detection. These samples, identified as: V05476, V06110, V05450, and V06106, and had CT values of 11.6, 14.3, 15.1, and 18.3, respectively. A total of 4.8 million reads were generated from the four samples with an N50 of 2,640 bases after 28 h of sequencing. The mean read coverage was approximately the same (7,529, 7,646, 7,673, and 7,725, respectively) for the four samples (Table 4). All the samples had high genome coverage (>98%), and each was assigned the BA.5 variant of Omicron. The number of reads mapped to each amplicon position is summarized in Figure 3 and Table 5. Out of seven amplicons, amplicon 4 had the highest number of reads mapped to the reference.

**Table 4:** Sequencing summary of four samples showing different quality metrics.

Sample	Raw reads	Filtered reads	Mapped to reference	Mean read coverage	Variant
V05476 _11.6	646,100	263,335	98.4%	7,529x	BA.5.1
V05450 _15.1	817,300	373,648	98.4%	7,646x	BA.5.2.1
V06110 _14.3	974,000	452,910	98.4%	7,672x	BA.5.3.1
V06106 _18.3	759,000	355,864	98.4%	7,725x	BA.5.2.1

**Table 5:** Total number of raw reads, filtered reads, and reads that mapped to the reference genome at seven amplicon regions.

Sample	V05476_11.6	V05450_15.1	V06110_14.3	V06106_18.3
Amplicon 1	24,006	39,741	53,725	33,702
Amplicon 2	24,656	30,882	51,645	34,150
Amplicon 3	34,223	45,931	51,497	46,092
Amplicon 4	36,750	57,256	84,273	48,869
Amplicon 5	22,471	32,822	31,826	29,876
Amplicon 6	21,170	24,926	21,167	31,987
Amplicon 7	26,924	42,728	41,629	40,237
Total mapped	190,200	274,286	335,762	264,913
Total raw reads	646.1 K	817.3 K	974 K	759.7 K
Filtered reads	263,335	373,648	452,910	355,864

**Figure 4:**
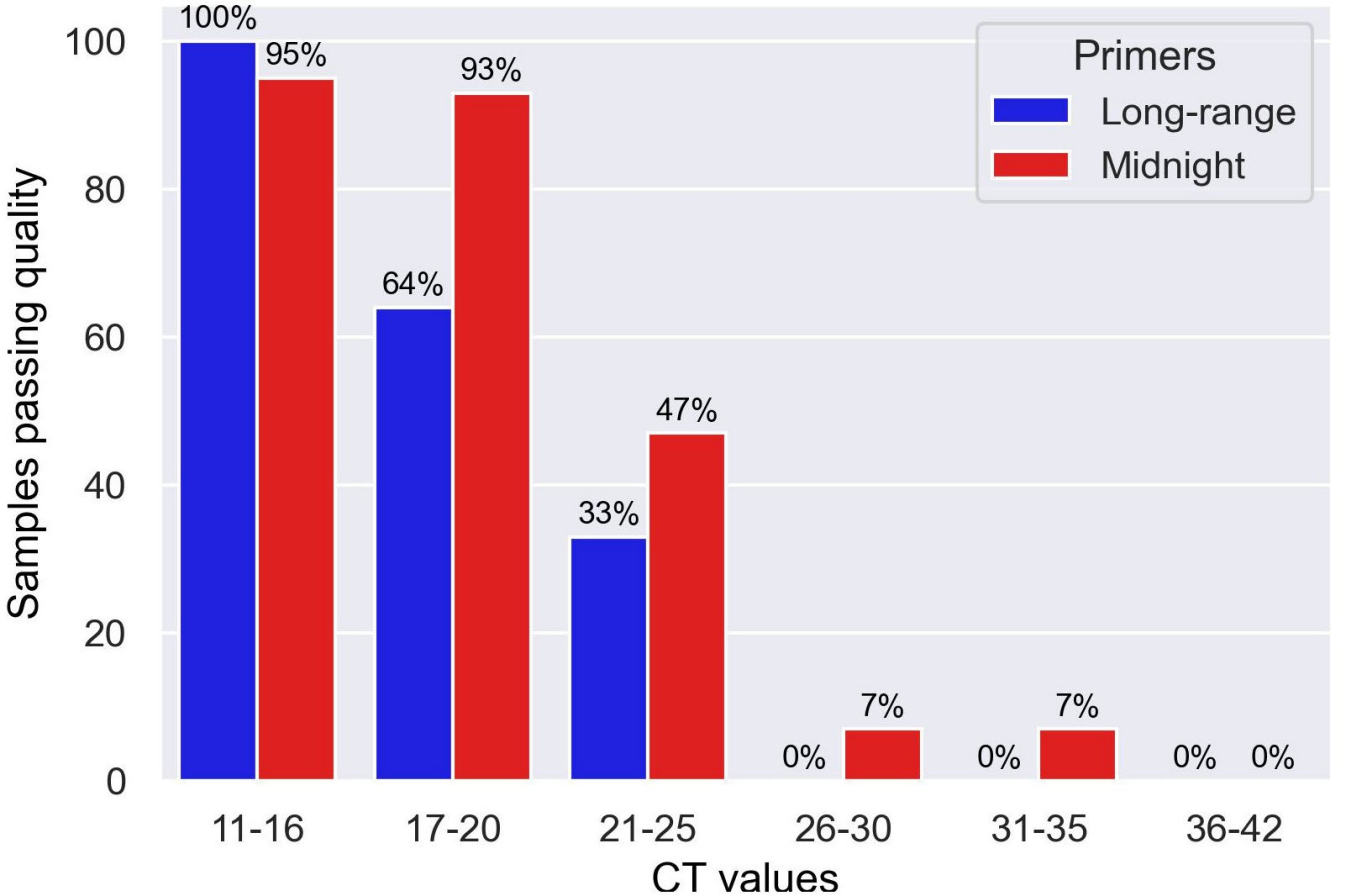
Bar chart showing samples sequenced with Midnight and Long-range primers with different CT values that passed quality.

A 96-well plate containing samples with different CT values spanning from 11 to 16 (*n* = 19), 17 to 20 (*n* = 14), 21 to 25 (*n* = 15), 26 to 30 (*n* = 15), 31 to 35 (*n* = 15), and 36 to 42 (*n* = 16) were sequenced using long-range and Midnight primers for comparison, as shown in Figure 3. With the long-range primers, 100% of the samples with CT values 11 to 16 passed quality, whereas 95% of samples within the range of this CT value passed quality when sequenced with midnight primers. Long-range primers performed similarly to the midnight primers for sequencing samples with CT values between 17 and 20 (Long-range: 73% and Midnight: 88% passing quality). For samples with CT values of 21–25, 47% passed quality with Midnight primers, whereas only 33% passed quality with long-range primers. With midnight primers, only two samples passed quality with CT values greater than 26. The long-range and the midnight primers generated no quality sequences in those samples with CT values greater than 26 (Figure 4 and Table 6). A heatmap of coverage for each amplicon regions is shown in the Supplementary Figures 2, 3.

**Table 6:** Comparison of total samples passing quality standards by CT values.

CT values	Samples	GenBank (Long-range)	GenBank (Midnight)
11–16	19	19 (100%)	18 (95%)
17–20	14	9 (73%)	13 (88%)
21–25	15	5 (33%)	7 (47%)
26–30	15	0	1 (0.07%)
31–35	15	0	1 (0.07%)
36–42	16	0	0
Total	94	32	40

Sample with different CT values were sequenced using Midnight primers and Long-range primers.

Although the samples from a 96-plex sequencing run that passed quality were accurately assigned to a lineage, we have found, in some cases, there was low coverage of some regions. Some of the amplicon regions such as 5, 6 and 7 had low coverage for some samples. We updated the primers specific to amplicon regions 5, 6 and 7 using the reference genomes of SARS-CoV-2 that were the most dominant during the time of writing this manuscript (Dec, 2022 to March, 2023). The updated primers were: 5-LEFT: 5ʹ-GTG ACT GGA CAA ATG CTG GTG A-3ʹ,5-RIGHT: 5’-CAT GAC ATA ACC ATC TAT TTG TTC GC-3ʹ, 6-LEFT: 5ʹ-GGT TCC GTG GCT ATA AAG ATA ACA G-3ʹ, 6-RIGHT: 5ʹ-AGG CTT GTA TCG GTA TCG TTG C-3ʹ, 7-LEFT: 5ʹ- GCC ATG GTA CAT TTG GCT AGG-3ʹ, 7-RIGHT: 5’-GCT CTT CCA TAT AGG CAG CTC-3ʹ. These alternative primers generated high-quality genomes with a lineage assigned to the consensus sequence of the genome (Supplementary Table 1). As the virus continues to mutate, it will likely be necessary to adjust the primers to maintain optimal coverage for all regions.

## 4 Discussion

We have developed and evaluated novel long-range primers to sequence SARS-CoV-2 clinical isolates using Oxford Nanopore sequencing. These novel primers can amplify regions approximately 4,500 base pairs. Using our primer set, the entire S-gene was sequenced using a single primer set. We compared the performance of long-range primers with midnight primers and found that long-range primers work as good as the midnight primers regarding the quality of genome sequences and coverage. This finding depends upon the amount of viral RNA in the sample. We decided to focus on comparison of the Midnight primers with the long-range PCR primers, and to exclude ARTIC primers, since much has already been published and discussed about the S-gene knockouts in the many short regions amplified by the ARTIC primers (Radhakrishnan et al., 2021; Carattini et al., 2023). Based on the results shown in Table 6, we can conclude that the optimal CT value for the long-range primers is 16 or lower, where we can consistently get 100% coverage. We can conclude that Midnight primers and long-range primers have better performance with CT values less than 20. Midnight primers are better than Long-range primers, however, long-range primers are as good as midnight primers with the advantage of having longer reads and the ability to minimize amplicon dropouts.

It is true that long-range primers are not better than midnight primers in generating high quality consensus sequences from clinical isolates of SARS-CoV-2 with very high CT values. However, long-range primers work as good as midnight primers for samples with CT values less than 20 as shown in Table 6 or Figure 4, Midnight primers generated 88% whereas long-range primers generate 73% of good quality genomes for GenBank. There are two advantages of long-range primers: first, this can solve the problems of amplicon dropouts due to mutations within primer binding site, and second, using single-molecule sequencing, these primers can be used to sequence across the entire S-gene region, and to assign lineages for population surveillance. Furthermore, the main objective of genomic surveillance during COVID-19 pandemic is to sequence as many genomes as possible with rapid and faster turnaround time.

We used 7,000 reference genomes from GISAID to generate a consensus sequence to design these long-range primers. Genome coverage is improved when primer schemes are created using multiple reference genome sequences compared to those designed using a single reference genome (Bei et al., 2022). ARTIC v3 and Midnight-1200 primers were designed using just one reference genome of SARS-CoV-2. In contrast, other primer schemes, such as the updated ARTIC (ARTIC v4.1), VarSkip Short v2, and VarSkip Long primers, were designed using multiple reference genomes. Long-range PCR primers can minimize the amplicon dropout due to mutations within the primer binding site (Bei et al., 2022).

After the ARTIC protocol was made public on January 22, 2020, these primers were adopted globally to sequence millions of SARS-CoV-2 genomes. After the introduction, there have been several improvements and updates to these primers to resolve dropouts and improve sequencing coverage (Grubaugh et al., 2019; Tyson et al., 2020; Davis et al., 2021). In addition to ARTIC primers, midnight primers that are extremely popular for sequencing SARS-CoV-2 clinical isolates using Nanopore sequencing were also updated to resolve amplicon dropouts and coverage bias along different regions of the viral genome (Constantinides et al., 2022). Several studies have been conducted to compare different sequencing protocols, using multiplex PCR primers to increase the genome coverage, improve the sequencing reading quality, eliminate amplicon dropouts, and improve coverage bias at different amplicon regions (Bei et al., 2022; Constantinides et al., 2022; Lambisia et al., 2022). As the virus mutates and spreads throughout communities, the primers and protocols need to be updated to avoid amplicon dropouts and avoid coverage bias. Because the S-gene is roughly 3,821 base pairs long, amplifying the entire S-gene requires multiple primer pairs using short-range primer pairs that are currently popular. Therefore, if any mutation occurs within the primer binding regions within S-gene, a significant fraction of S-gene could be dropped from final consensus sequence.

Long-range primers to sequence SARS-CoV-2 have previously not been reported, apart from a few primer schemes amplifying regions up to 2,500 base pairs (Arana et al., 2022). Because the S-gene is approximately 3,821 base pairs, amplifying the entire S-gene requires more than one primer. Therefore, mutations within S-gene could result in dropout within S-gene. As an alternative to this problem, leveraging the long-read sequencing available with Oxford Nanopore flow cells, we have developed long-range primers, which sequence the entire S-gene using just one primer pair, thereby eliminating the possibility of amplicon dropout due to mutations within S-gene.

The accuracy of PCR reactions for the long-range primers depends upon many factors, including the specificity of the primers, experimental conditions such as number of PCR cycles, cycle parameters, and environmental conditions such as Mg^2+^ concentrations, and which DNA polymerases are used for amplification. For the purposes of the experiments outlined here, ‘accuracy’ is important in terms of obtaining full length cDNA sequences for the segments of interest. In principle, we could quantitate the transcripts using something like droplet digital PCR (ddPCR; Hindson et al., 2013; Kojabad et al., 2021). However, the main purpose of this current work is to reduce the number of primers used, and to verify that we are indeed getting full length sequences of the regions of interest (which is verified by sequence alignment).

A limitation of this approach is that a mutation within the primer binding sites can result in a drop out of that entire region, leading to a more significant gap in the consensus sequence that significantly affects the quality of the genome sequence. However, since the primer sites were designed using conserved regions, we anticipate that this will continue to work, although, as necessary, it is easy to update the primers for novel strains. Another limitation is associated with viral load in the sample. We have found that although these long-range primers can amplify larger segments of the viral genome, these primers are not well suited to sequence samples with higher CT values (greater than 25).

Single-read sequencing technologies could quantitate viral genome fragmentation. For example, the viral genome is sheared into smaller pieces, most of the sequence reads would be short (and little amplification would occur, since this would be essentially linear extension of only one strand). We have designed primers in well conserved regions, that will hopefully have few mutations. One of the advantages of long-range primers is that fewer primer sites are necessary, and also more flexibility is given with respect to the placement of the primers, allowing for better optimization of primer binding sites within conserved regions.

Although WHO lifted the global health emergency due to a significant reduction in positive cases, we are entering into a new phase of COVID-19 as 1 out of 10 people have long-haul COVID (Thaweethai et al., 2023). Looking back to historical epidemics due to coronavirus and the evolutionary relatedness of the SARS-CoV-2 with previous outbreaks of SARS and MERS, future pandemics are inevitable. COVID-19 is still circulating as local outbreaks continue. The long-range PCR method outlined here can help with surveillance of community infections through wastewater monitoring. With single reads over the entire S-gene region, it is possible to quantitate variant diversity within a sample. This will allow monitoring of emerging variants as well as keeping track of known variants of concern.

**Table 7:** GenBank accession number of the samples used to validate this study’s long-range primers.

Sample_ID	GenBank accession
V05450_15.1	OP576060
V06110_14.3	OQ079743
V06106_18.3	OQ079740
V06501_12.1	OQ938311
V06507_12.7	OQ938315
V06508_12.4	OQ938316

The samples used in this study were previously sequenced using either Midnight primers in Oxford Nanopore or using ARTIC primers in Illumina.

In conclusion, in this study we have shown the applications of long-range primers to sequence SARS-CoV-2 mainly in the context of surveillance to address the issues of amplicon dropouts as observed by using primers that amplify short regions. While the long-range primers might be affected by the quality of the viral genetic material, we have shown that we can sequence the most important region of the virus using just one primer set (flanking the S-gene) which would help to determine the emerging variants.

## Data availability statement

The datasets presented in this study can be found in online repositories. The names of the repository/repositories and accession number(s) can be found in the article/Supplementary material. The datasets analyzed in this study are deposited in NCBI, accession numbers: OP576060, OQ079743, OQ079740, OQ938311, OQ938315, OQ938316 (Table 7).

## Author contributions

SK: Conceptualization, Data curation, Formal analysis, Investigation, Methodology, Software, Validation, Visualization, Writing – original draft, Writing – review & editing. SH: Methodology, Writing – review & editing. AI: Methodology, Writing – review & editing. GT: Methodology, Writing – review & editing. JK: Funding acquisition, Project administration, Resources, Supervision, Writing – review & editing. DU: Conceptualization, Funding acquisition, Project administration, Resources, Supervision, Validation, Writing – review & editing.

## Footnotes

^1^https://gisaid.org/

^2^https://www.ncbi.nlm.nih.gov/sars-cov-2/

^3^https://covariants.org

^4^https://www.ncbi.nlm.nih.gov/projects/msaviewer/

^5^https://artic.network/ncov-2019/ncov2019-bioinformatics-sop.html

## Supplementary material

SUPPLEMENTARY FIGURE 1 IGV plot showing mutations at position 27,897 of a Delta variant sample sequenced in Nanopore using Midnight primers. This mutation occurs within the primer binding region for the amplicon 28 (28_LEFT). This is one of the early dropouts observed in most genome sequences generated using Midnight primers.

SUPPLEMENTARY FIGURE 2 Heatmap of 94 SARS-CoV-2 samples sequenced using Long-range primers. Dark color intensity represents high numbers of reads, and light regions represents low amplicon coverage.

SUPPLEMENTARY FIGURE 3 Heatmap of 94 SARS-CoV-2 samples sequenced using Midnight primers. Dark color intensity represents high numbers of reads, and light regions represents low amplicon coverage.
