# Supplementary material for: Genomic surveillance of SARS-CoV-2 using long-range PCR primers
Source: Front Microbiol. 2024 Feb 14;15:1272972. doi: 10.3389/fmicb.2024.1272972 (PMC10910555; doi:10.3389/fmicb.2024.1272972)
Supplement: Supplementary file 1 [file Table_1.docx]

**Supplementary Table 1**: Nextclade results for three samples sequencing using updated long-range primers. The samples had a CT value of 12.

| Sample | Clade | Pango lineage | Mutations | Read coverage | Ns | Coverage | Gaps |
| --- | --- | --- | --- | --- | --- | --- | --- |
| V06501_12.1 | 22B | BW.1 | 76 | 1349x | 345 | 98.8% | 36 |
| V06507_12.7 | 22B | BA.5.2.1 | 81 | 2701x | 278 | 99.1% | 33 |
| V06508_12.4 | 22E | BQ.1.1 | 78 | 2339x | 251 | 99.2% | 33 |
