# Supplementary figures and images for: Genomic surveillance of SARS-CoV-2 using long-range PCR primers
Source: Front Microbiol. 2024 Feb 14;15:1272972. doi: 10.3389/fmicb.2024.1272972 (PMC10910555; doi:10.3389/fmicb.2024.1272972)

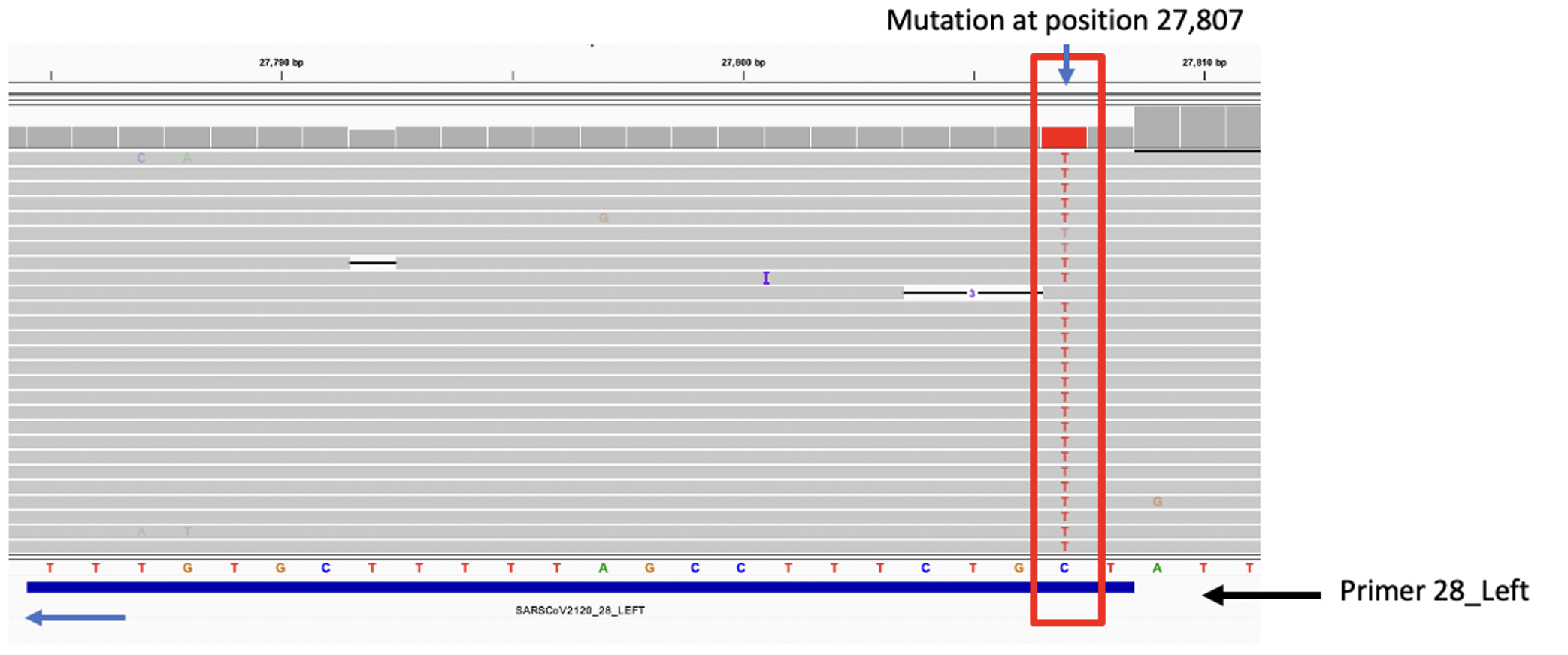

Supplement: SUPPLEMENTARY FIGURE 1 — IGV plot showing mutations at position 27,897 of a Delta variant sample sequenced in Nanopore using Midnight primers. This mutation occurs within the primer binding region for the amplicon 28 (28_LEFT). This is one of the early dropouts observed in most genome sequences generated using Midnight primers. [file Image_1.TIFF]

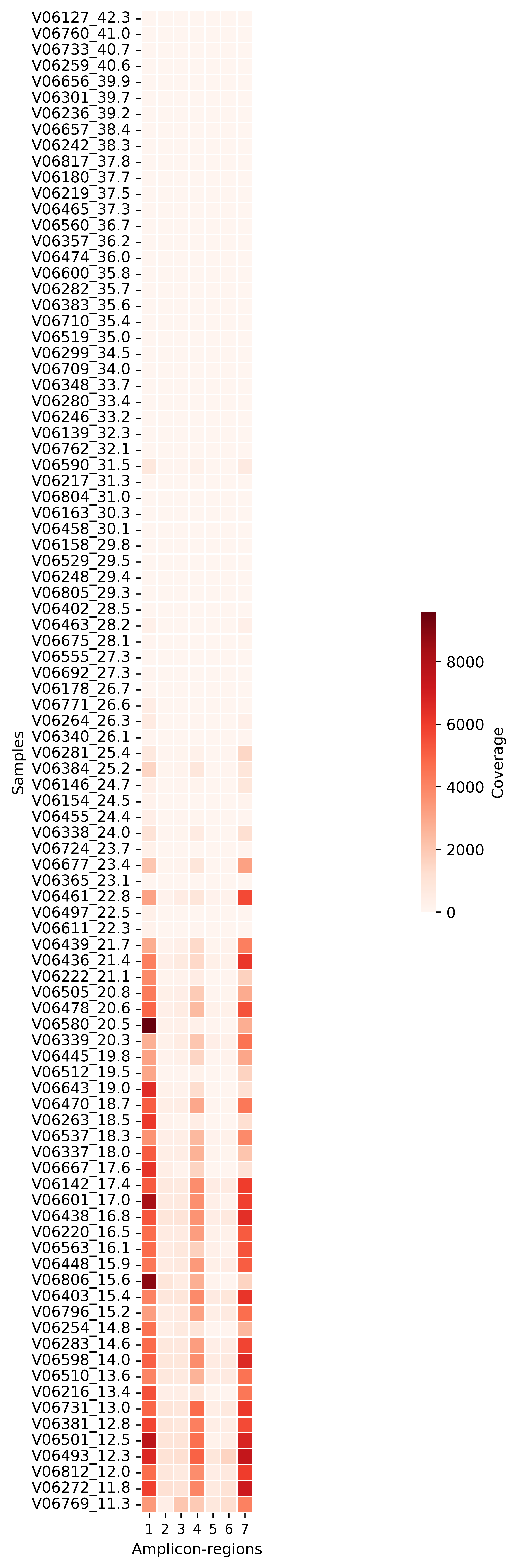

Supplement: SUPPLEMENTARY FIGURE 2 — Heatmap of 94 SARS-CoV-2 samples sequenced using Long-range primers. Dark color intensity represents high numbers of reads, and light regions represents low amplicon coverage. [file Image_2.TIF]

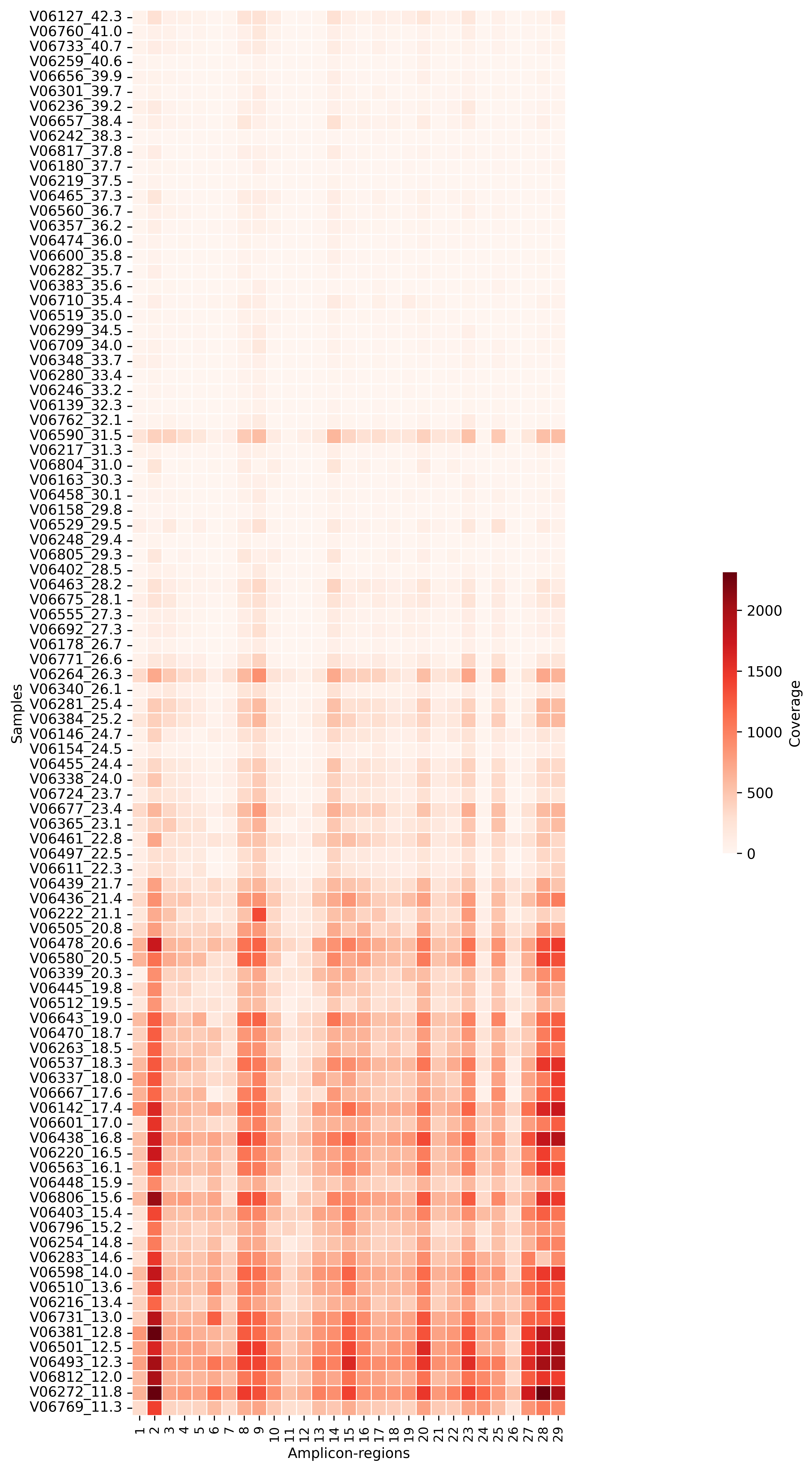

Supplement: SUPPLEMENTARY FIGURE 3 — Heatmap of 94 SARS-CoV-2 samples sequenced using Midnight primers. Dark color intensity represents high numbers of reads, and light regions represents low amplicon coverage. [file Image_3.TIF]
